# Associations Between Physical Activity, Sedentary Behaviour, Sleep Quality, and Anxiety Among Healthy Pain-Free Adults in Saudi Arabia: An Online Cross-Sectional Survey

**DOI:** 10.3390/sports13070196

**Published:** 2025-06-20

**Authors:** Mansour Abdullah Alshehri, Areej Ali Alzylai, Joree Abdulrahman Alshehri, Lana Zaid Alsharif, Wafa Saleem Almalki, Hammad S. Alhasan, Ammar S. Fadil, Moayad Saleh Subahi, Hosam Alzahrani

**Affiliations:** 1 Department of Medical Rehabilitation Sciences, Faculty of Applied Medical Sciences, Umm Al-Qura University, Mecca 24382, Saudi Arabia; s442013070@uqu.edu.sa (A.A.A.); s443002163@uqu.edu.sa (J.A.A.); s442006878@uqu.edu.sa (L.Z.A.); s443011880@uqu.edu.sa (W.S.A.); hshasan@uqu.edu.sa (H.S.A.); asfadil@uqu.edu.sa (A.S.F.); mssubahi@uqu.edu.sa (M.S.S.); 2 Department of Physical Therapy, College of Applied Medical Sciences, Taif University, Taif 21994, Saudi Arabia; halzahrani@tu.edu.sa

**Keywords:** physical activity, sedentary behaviour, GPAQ, sleep quality, B-PSQI, anxiety, GAD-7, healthy adults, Saudi Arabia, cross-sectional study

## Abstract

Physical activity, sedentary behaviour, sleep quality, and anxiety are interrelated factors that play a critical role in physical and mental health. However, limited evidence exists on their associations within the Saudi Arabian context. This study aimed to investigate the relationships between physical activity, sedentary behaviour, sleep quality, and anxiety symptoms among healthy, pain-free adults in Saudi Arabia. A cross-sectional online survey was conducted between December 2024 and April 2025 using validated Arabic versions of the Global Physical Activity Questionnaire (GPAQ), the Brief Pittsburgh Sleep Quality Index (B-PSQI), and the Generalised Anxiety Disorder-7 (GAD-7). The data were analysed using descriptive statistics and multivariable linear regression models. A total of 185 participants (mean age = 33.99 ± 12.57 years; 69.73% female) were included. The average total physical activity was 1730.02 ± 2109.24 MET min/week, and the mean sedentary time was 6.77 ± 4.69 h/day. No significant associations were found between physical activity and either sleep quality or anxiety symptoms. Similarly, sedentary behaviour time was not significantly associated with sleep quality or anxiety symptoms. However, poorer sleep quality was significantly associated with higher anxiety levels. These findings highlight the importance of sleep quality in mental health strategies for healthy adults in Saudi Arabia.

## 1. Introduction

Physical activity, sedentary behaviour, sleep quality, and anxiety are interconnected domains that significantly influence both physical and mental health. Regular physical activity is well-documented to reduce the risk of cardiovascular disease, type 2 diabetes, obesity, and mental health conditions such as depression and anxiety [1,2]. Nonetheless, physical inactivity remains a global concern, with the World Health Organization (WHO) estimating that 23% of adults are insufficiently active [3]. In Saudi Arabia, inactivity and sedentary behaviour are particularly widespread. National data indicate that 77% of adults were inactive before the COVID-19 lockdown, and over 60% remained inactive during restrictions [4]. Among university students, inactivity rates exceed 60%, especially among females [5,6], and over 85% of female students report sedentary time exceeding three h per day [6]. These patterns are influenced by structural and sociocultural barriers, including limited infrastructure, urban design, and restrictive norms [7]. Although independent of physical activity, prolonged sedentary behaviour has also been associated with adverse health outcomes [8].

Sleep quality is a multidimensional construct that includes sleep latency, duration, continuity, and subjective satisfaction [9]. Good quality sleep is essential for cognitive function, emotional regulation, immune health, and metabolic processes [10,11]. Chronic sleep disturbances have been linked to an increased risk of hypertension, obesity, type 2 diabetes, depression, and impaired quality of life [12]. It is estimated that 50–70 million adults worldwide suffer from a sleep disorder [13]. In Saudi Arabia, poor sleep quality has been widely documented across various population groups, including university students, healthcare workers, and the general adult population, with prevalence estimates ranging from 52% to 76% [5,14,15,16,17].

Anxiety disorders, characterised by excessive worry and physiological hyperarousal, are among the most common mental health conditions globally, affecting approximately 264 million individuals [18]. In Saudi Arabia, the prevalence of anxiety has increased in recent years, with estimates ranging from 12% to over 40% across different populations, including university students, working adults, healthcare workers, and the general public [7,16,19,20].

An increasing body of literature supports a bi-directional relationship between physical activity, sedentary behaviour, sleep quality, and anxiety symptoms, underpinned by both physiological and psychological mechanisms [9,21,22,23,24,25,26]. Regular physical activity can improve sleep quality by helping to regulate the body’s internal clock, increase sleep pressure, lower body temperature after exercise, and boost melatonin levels. These changes help people fall asleep faster and stay asleep longer [22,24,25]. Physical activity also helps reduce anxiety symptoms by increasing the release of chemicals in the brain that improve mood, such as endorphins, serotonin, and dopamine [27]. It may also reduce stress levels by lowering the activity of the stress-response system and supporting better balance in the nervous system, which together improve both sleep and mood [24,28]. On the other hand, poor sleep quality and high levels of anxiety symptoms can lead to low energy and reduced motivation, making people more likely to spend time sitting and less likely to be active [21,26,29]. Despite these well-established interrelationships, limited research has explored their combined effects in healthy adult populations, particularly within the Arab context.

In Saudi Arabia, an increasing number of studies have examined the associations among physical activity, sedentary behaviour, sleep quality, and anxiety symptoms across different populations. Several of these studies have reported significant pairwise associations, such as between physical activity and sleep quality [5,16,17,30], physical activity and anxiety symptoms [4,7], sedentary behaviour and sleep quality [6], sedentary behaviour and anxiety symptoms [7], and sleep quality and anxiety symptoms [14,15,16,19,20]. However, existing research has primarily investigated these associations in isolation, without concurrently addressing the full set of interrelationships among physical activity, sedentary behaviour, sleep quality, and anxiety symptoms. To date, no study has simultaneously included all these variables in a single study to gain better insight into their potential bidirectional relationships. Importantly, no previous research has specifically examined these associations in healthy adults who are free from musculoskeletal pain and any medical conditions. Studying this subpopulation allows for the reduction in potential clinical confounders and may improve the validity and interpretability of observed associations. This research gap is particularly relevant given the consistently high prevalence of physical inactivity, prolonged sedentary time, poor sleep quality, and anxiety symptoms in Saudi Arabia, all of which are influenced by cultural, environmental, and infrastructural factors [6,7]. Therefore, the aim of the present study was to investigate the associations among physical activity, sedentary behaviour, sleep quality, and anxiety symptoms in a sample of healthy, pain-free adults residing in Saudi Arabia. A more comprehensive understanding of these interrelated variables may support the development of targeted, culturally appropriate public health strategies to enhance both physical and mental well-being in this population.

## 2. Materials and Methods

### 2.1. Study Design and Ethics

This study adopted a cross-sectional survey design to examine the relationships between physical activity, sleep quality, and anxiety. A convenience sampling method was employed, and data were collected through an online survey to maximise accessibility and promote wide participation. Informed consent was obtained electronically, with comprehensive information provided on the first page of the survey outlining the study’s purpose, procedures, confidentiality assurances, and the voluntary nature of participation. Ethical approval for the study was obtained from the Biomedical Research Ethics Committee at Umm Al-Qura University, Mecca, Saudi Arabia (Approval Number: HAPO-02-K-012-2024-12-2352). This study was conducted in accordance with the Strengthening the Reporting of Observational Studies in Epidemiology (STROBE) guidelines (Table S1) and includes all required reporting components.

### 2.2. Sample Size Estimation

A post hoc sample size analysis was conducted using G*Power (version 3.1.9.7; Universität Düsseldorf, Düsseldorf, Germany) to evaluate whether the final sample size was adequate for the multivariable regression analyses performed in the study. The analysis included five models examining the associations between physical activity, sedentary behaviour, sleep quality, and anxiety symptoms. Sample size was calculated using the “linear multiple regression: fixed model, R^2^ deviation from zero” procedure. The calculation assumed a medium effect size (f^2^ = 0.15), a significance level of 0.05, power of 0.95, and 11 predictors per model (including the main independent variable and 10 covariates/factors). Based on these parameters, the minimum required sample size was estimated at 178 participants. As the final sample exceeded this threshold, the study was considered adequately powered to investigate the anticipated associations.

### 2.3. Eligibility Criteria

Eligible participants were healthy, pain-free adults aged 18 years or older residing in Saudi Arabia. Individuals of all nationalities were considered for inclusion. The exclusion criteria were as follows: (1) self-reported musculoskeletal pain within the preceding week; (2) the presence of any self-reported clinically significant medical conditions, including but not limited to: hypertension, diabetes mellitus, asthma, anaemia, dyslipidaemia, urinary incontinence, chronic kidney disease, chronic obstructive pulmonary disease, coronary artery disease, arthritis (e.g., osteoarthritis, rheumatoid arthritis), osteoporosis, cancer, neurological disease (e.g., stroke, multiple sclerosis), chronic musculoskeletal pain at any site, history of serious fractures (e.g., hip or femur fractures), or history of any major surgery (e.g., total knee replacement, spinal fusion, coronary artery bypass grafting); (3) incomplete responses to any of the four primary sections of the survey (as outlined below); and (4) failure to provide informed consent via the initial page of the online survey.

### 2.4. Data Collection

Data for this study were collected through a self-administered online survey hosted on the SurveyMonkey platform (www.surveymonkey.com). The survey incorporated validated Arabic versions of the physical activity, sleep quality, and anxiety questionnaires to ensure both linguistic accuracy and cultural relevance. It targeted adult residents of Saudi Arabia and was available for a five-month period, from December 2024 to April 2025. Recruitment was conducted using digital posters and advertisements featuring a QR code and direct survey link to facilitate participant access. These materials were distributed via multiple online platforms, including X (formerly Twitter), Facebook, and WhatsApp. All responses were submitted anonymously, and no personally identifiable information was collected. Access to the dataset was restricted solely to the research team to maintain participant confidentiality and ensure compliance with ethical data management standards.

### 2.5. Survey Structure

The survey comprised four primary sections: (1) demographic information, which included individual characteristics and questions related to eligibility criteria; (2) the Global Physical Activity Questionnaire (GPAQ), used to assess participants’ physical activity volume; (3) the Brief Version of the Pittsburgh Sleep Quality Index (B-PSQI), designed to evaluate sleep quality; and (4) the Generalised Anxiety Disorder-7 (GAD-7) scale, used to assess mental health, specifically symptoms of anxiety. The GPAQ [31,32], PSQI [33,34,35], and GAD-7 [36,37,38,39] were administered in Arabic using previously validated versions. These instruments have demonstrated acceptable psychometric properties in Arabic-speaking populations. The Arabic version of the GPAQ showed good test–retest reliability, with correlation coefficients ranging from 0.62 to 0.78 across different physical activity domains [32]. The internal consistency of the Arabic PSQI was moderate, with Cronbach’s alpha values reported between 0.60 and 0.65 in various studies [33,34,35]. For the Arabic GAD-7, Cronbach’s alpha values ranged from 0.76 to 0.95, indicating good to excellent internal consistency [37,38], and test–retest reliability was also adequate, with an intraclass correlation coefficient of 0.83 [39].

#### 2.5.1. Demographic Information and Eligibility Screening

The first section of the survey collected detailed demographic and eligibility-related data to contextualise the study findings and control for potential confounding variables. Participants were asked to provide information on key personal characteristics, including age, gender, height, weight, nationality, region of residence, living arrangement, marital status, educational attainment, current employment status, primary work environment, average number of work or study hours per day, and smoking status. To determine eligibility, this section also incorporated screening items related to musculoskeletal health and medical history (see Section 2.3). Specifically, participants were asked whether they had experienced any musculoskeletal pain in the previous week and whether they had a history of significant injuries or surgeries. Additionally, participants were screened for the presence of serious or chronic medical conditions. Only adults aged 18 years or older who reported being pain-free and free of major injuries or chronic health conditions were included in the final analysis.

#### 2.5.2. Physical Activity and Sedentary Behaviour

Physical activity was assessed using the GPAQ, a standardised and validated instrument developed by the World Health Organization (WHO) for monitoring physical activity across diverse populations [40]. The GPAQ captures physical activity across three distinct domains: (1) work-related activity, (2) travel to and from places (transport), and (3) recreational activities [40]. For each domain, participants were asked to report the number of days per week and the duration in min per day they engaged in moderate- and vigorous-intensity physical activities. In accordance with WHO analytical guidelines, responses from all three domains were used to compute the total weekly volume of physical activity, expressed in metabolic equivalent task (MET) min per week [41]. The total MET min per week was computed using the following equation:

*Total MET min*/*week* = (*Vigorous Work Days* × *Minutes* × 8.0) + (*Moderate Work Days* × *Minutes* × 4.0) + (*Transport Days* × *Minutes* × 4.0) + (*Vigorous Recreation Days* × *Minutes* × 8.0) + (*Moderate Recreation Days* × *Minutes* × 4.0).

Participants were subsequently classified based on the WHO recommendations for physical activity. Those who achieved a total of at least 600 MET min per week were considered to have met the recommended threshold for achieving health-enhancing levels of physical activity [1]. This benchmark is associated with a reduced risk of noncommunicable diseases and improved overall health outcomes. All physical activity data were screened for plausibility in accordance with the GPAQ data processing guidelines. Participants reporting implausible activity durations (e.g., total activity exceeding 24 h per day) or unusually high total MET-min per week were identified for exclusion. In addition to physical activity, the average duration of sedentary behaviour (in hours per day) was also calculated based on participants’ responses to the GPAQ, providing further insight into their daily activity patterns.

#### 2.5.3. Sleep Quality

Sleep quality was assessed using the B-PSQI, a psychometrically validated self-report instrument derived from the original PSQI [42]. It includes six items capturing five components of sleep: bedtime and wake-up time (used to compute sleep efficiency), sleep latency, total sleep duration, night awakenings, and subjective sleep quality [43]. Each item is scored on a 4-point scale ranging from 0 (no difficulty or very good sleep) to 3 (severe difficulty or very poor sleep), yielding a global score from 0 to 15. Higher scores indicate poorer sleep quality. A total score >5 is used as a validated cutoff to identify individuals with poor sleep quality [43].

#### 2.5.4. Anxiety Symptoms

Anxiety symptoms were assessed using the GAD-7 scale, a widely utilised and psychometrically validated screening instrument for evaluating the severity of generalised anxiety symptoms [44,45]. The GAD-7 comprises seven items that measure how frequently participants have been bothered by anxiety-related problems. Each item is rated on a 4-point Likert scale ranging from 0 (“not at all”) to 3 (“nearly every day”), yielding a total score ranging from 0 to 21. According to established thresholds, total scores are interpreted as follows: 0–4 (minimal anxiety), 5–9 (mild anxiety), 10–14 (moderate anxiety), and 15–21 (severe anxiety) [44].

### 2.6. Data Analysis

Responses that included only demographic information but lacked completion of the primary survey components (GPAQ, B-PSQI, and GAD-7) were excluded from the final analysis. All statistical analyses were performed using StataMP, version 17 (StataCorp LLC, College Station, TX, USA). A *p*-value of less than 0.05 was considered statistically significant for all analyses conducted.

Descriptive statistics, including frequencies and percentages, were used to summarise participant characteristics and response distributions. Additionally, mean values and standard deviations (SD) were calculated to quantify total MET min per week for physical activity and sedentary behaviour time (in hours), both derived from the GPAQ, as well as overall scores for sleep quality (B-PSQI) and anxiety (GAD-7). To further explore associations between categorical variables, a cross-tabulation with a chi-square test was conducted to examine the distribution of anxiety severity (minimal, mild, moderate, and severe) across sleep quality categories (good and poor).

Multivariable linear regression analyses were conducted to examine the associations between (1) physical activity and sleep quality, (2) physical activity and anxiety symptoms, (3) sedentary behaviour time and sleep quality, (4) sedentary behaviour time and anxiety symptoms, and (5) sleep quality and anxiety symptoms. Each model was adjusted for a set of potential confounding variables, including age, gender, body mass index (BMI), nationality, region of residence, marital status, educational attainment, current employment status, primary work environment, and smoking status. These covariates/factors were included to control for their potential influence on the outcomes and to ensure more accurate estimation of the associations of interest. To further explore whether the association between physical activity and the outcomes (sleep quality and anxiety symptoms) varied by activity level, additional interaction analyses were conducted. Specifically, interaction terms between total physical activity (MET-min/week) and a binary indicator of meeting the WHO physical activity recommendation (≥600 MET-min/week) were included in multivariable models for sleep quality and anxiety symptoms. These models were similarly adjusted for all covariates/factors. Residual diagnostics were conducted for each model to assess the normality assumption. While minor deviations from normality were noted, the sample size was sufficient for linear regression to remain robust under these conditions. To further account for potential heteroskedasticity and mild non-normality of residuals, all regression models were estimated using robust standard errors. For each model, the regression coefficient (β) and its 95% confidence interval (CI) were reported to indicate the direction and strength of the associations, along with the R-squared (R^2^) value to reflect the proportion of variance explained by the model.

## 3. Results

### 3.1. Participant Selection

A total of 322 participants who reported no musculoskeletal pain were initially considered for inclusion. Of these, 94 participants were subsequently excluded: 16 due to inconsistent responses indicating both the presence and absence of musculoskeletal pain and 78 due to self-reported medical conditions, including hypertension, diabetes mellitus, asthma, coronary artery disease, hyperlipidaemia, anaemia, chronic kidney disease, vertigo, cancer, recent major surgery, urinary incontinence, bone fractures, stroke, or gastrointestinal disorders. This exclusion process yielded a subsample of 228 participants who met the health-related eligibility criteria. An additional 43 participants were excluded due to incomplete responses in one or more of the four primary sections of the survey. The final analytical sample therefore comprised 185 healthy, pain-free adults with complete data across all components of the questionnaire.

### 3.2. Demographic Information

The final analytical sample comprised 185 participants with a mean age of 33.99 years (SD = 12.57). The majority were female (69.73%) and Saudi nationals (91.35%). The mean BMI was 26.30 kg/m^2^ (SD = 5.53), indicating an overall classification within the overweight range. Participants predominantly resided in the Mecca region (54.05%) and the Riyadh region (20.00%), with lower representation from other regions. Approximately half of the participants were single (50.81%), while 44.32% were married. In terms of educational attainment, 41.08% of participants were either currently enrolled in or had completed a bachelor’s degree, while 22.16% were pursuing or had completed postgraduate studies at the master’s or doctoral level. Regarding employment status, 44.32% of participants were employed full-time, while 26.49% were students not currently engaged in employment. The most frequently reported work setting was office- or desk-based (42.70%), followed by standing or on-foot occupations (36.76%). The majority of participants identified as never having smoked (70.81%), while 13.51% were current smokers. Further demographic characteristics are presented in Table 1.

### 3.3. Physical Activity, Sedentary Behaviour, Sleep Quality, and Anxiety Symptoms

The participants reported a wide range of physical activity, with a mean total of 1730.02 ± 2109.24 MET min per week, as calculated from the GPAQ, indicating substantial variability in physical activity volume. Sedentary behaviour, also derived from the GPAQ, averaged 6.77 ± 4.69 h per day, reflecting considerable differences in daily sitting and reclining time across the sample. The average score for sleep quality, as measured by the B-PSQI, was 4.05 ± 2.75, while the mean anxiety level based on the GAD-7 was 4.16 ± 3.66, suggesting generally mild symptoms on average. These summary statistics are presented in Table 2.

**Figure 1:**
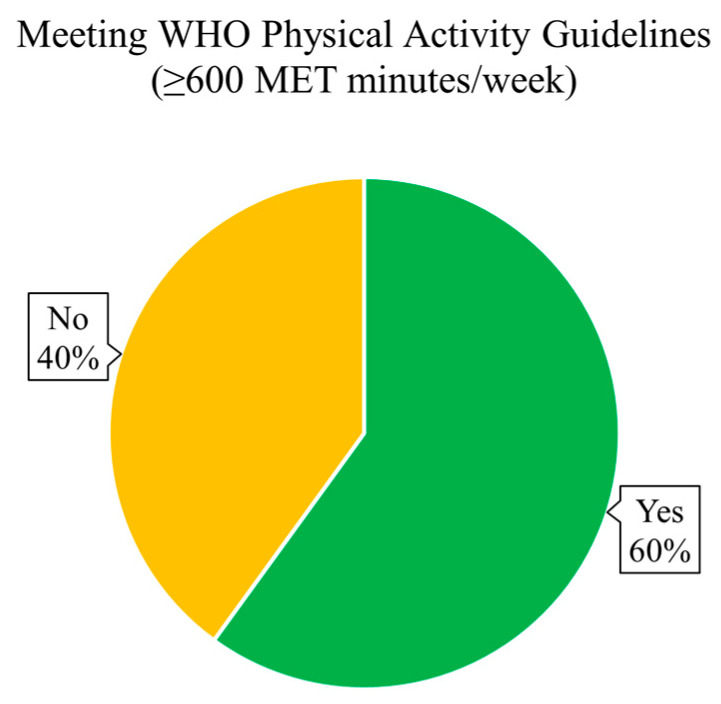
Proportion of participants meeting WHO physical activity guidelines. A pie chart showing the distribution of participants based on the WHO’s physical activity recommendation (≥600 MET min/week) based on GPAQ.

**Figure 2:**
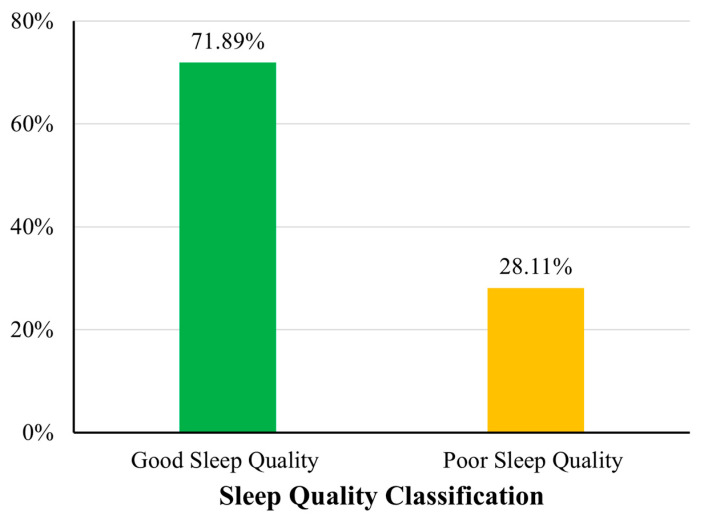
Sleep quality classification based on B-PSQI scores. A bar chart showing the proportion of participants categorised by sleep quality.

**Figure 3:**
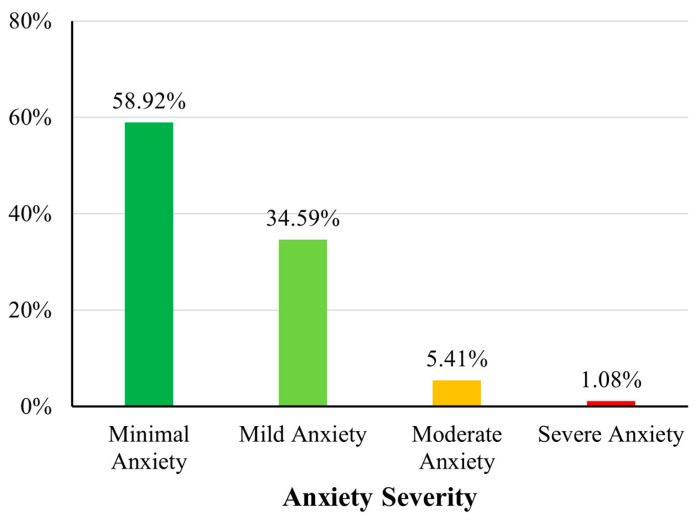
Distribution of anxiety severity levels based on GAD-7 categories. A bar chart showing the distribution of participants across GAD-7 anxiety severity categories.

Based on the WHO physical activity guidelines (≥600 MET min/week), 60% (n = 111) of participants met the recommended activity threshold, whereas 40% (n = 74) did not (Figure 1). Regarding sleep quality, 28.11% (n = 52) of participants were classified as having poor sleep quality (B-PSQI > 5), while the remaining 71.89% (n = 133) reported good sleep quality (Figure 2). Anxiety severity levels, as categorised using the standard GAD-7 cutoffs, revealed that 58.92% (n = 109) had minimal anxiety, 34.59% (n = 64) had mild anxiety, 5.41% (n = 10) had moderate anxiety, and 1.08% (n = 2) reported severe anxiety symptoms (Figure 3).

A cross-tabulation of anxiety severity and sleep quality categories revealed no statistically significant association (*p* = 0.163). While the majority of participants with minimal anxiety reported good sleep quality (77.98%), a relatively higher proportion of those with mild to severe anxiety reported poor sleep quality; however, these differences did not reach statistical significance (Table 3).

### 3.4. Associations Between Physical Activity, Sleep Quality, and Anxiety Symptoms

**Figure 4:**
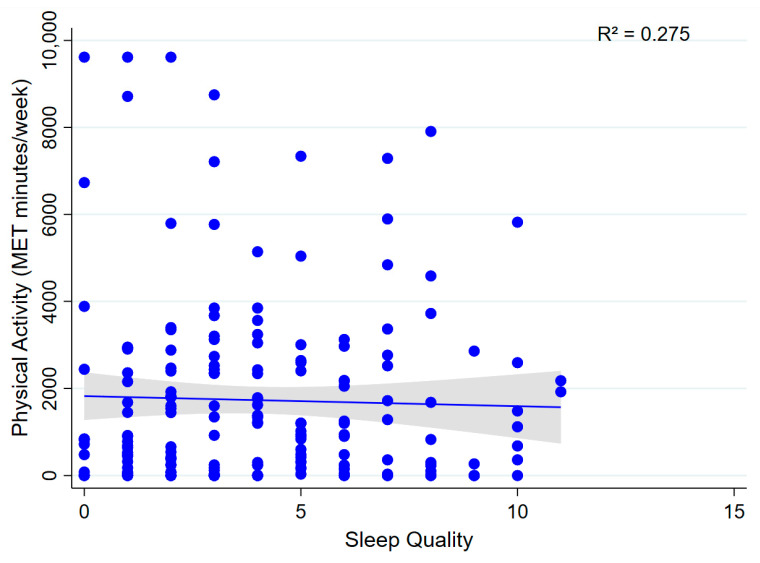
Association between physical activity and sleep quality. A scatter plot showing the relationship between total physical activity (MET min/week) and sleep quality.

**Figure 5:**
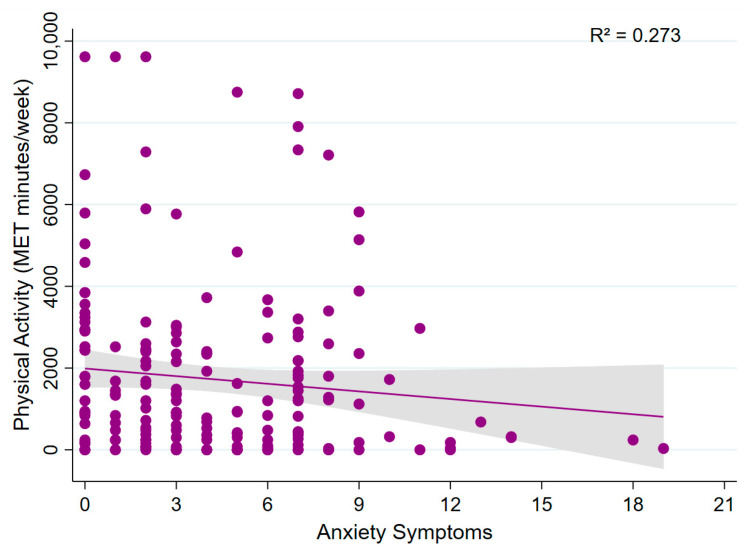
Association between physical activity and anxiety symptoms. A scatter plot showing the relationship between total physical activity (MET min/week) and anxiety symptoms.

Multivariable linear regression analyses showed that total physical activity (MET min/week) was not significantly associated with sleep quality (β = −70.80; 95% CI: −203.04 to 61.44; *p* = 0.292; model R^2^ = 0.275; Figure 4) or anxiety symptoms (β = −46.96; 95% CI: −125.74 to 31.82; *p* = 0.241; model R^2^ = 0.273; Figure 5). These findings suggest that, within this sample, physical activity levels were not significantly associated with either sleep quality or anxiety symptoms.

Interaction analyses revealed no significant moderation effects of meeting WHO physical activity guidelines on the relationship between total physical activity and the outcomes. For sleep quality, the interaction term was not significant (β = 0.002; 95% CI: −0.002 to 0.006; *p* = 0.314; model R^2^ = 0.220). Similarly, for anxiety symptoms, the interaction effect was non-significant (β = 0.006^−2^; 95% CI: −0.006 to 0.006; *p* = 0.983; model R^2^ = 0.282). These findings indicate that the associations between physical activity and both sleep quality and anxiety symptoms did not differ based on whether participants met WHO physical activity recommendations.

### 3.5. Associations Between Sedentary Behaviour, Sleep Quality, and Anxiety Symptoms

**Figure 6:**
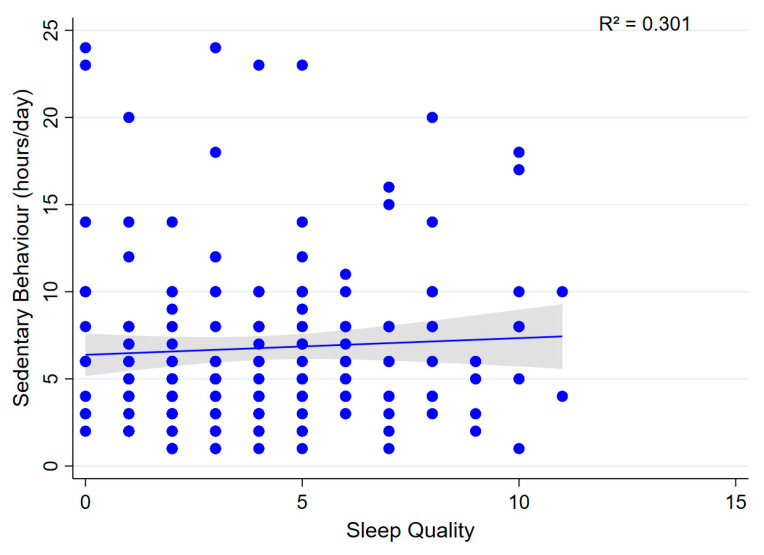
Association between sedentary behaviour and sleep quality. A scatter plot showing the relationship between sedentary behaviour (h/day) and sleep quality.

**Figure 7:**
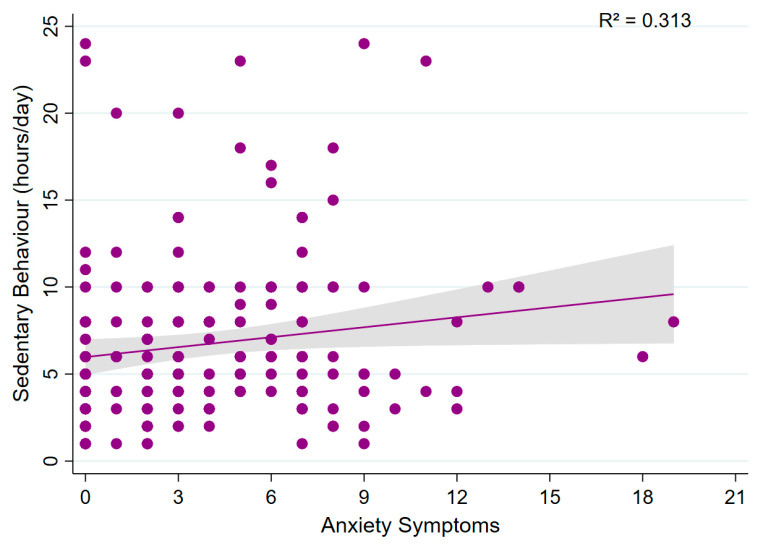
Association between sedentary behaviour and anxiety symptoms. A scatter plot showing the relationship between sedentary behaviour (h/day) and anxiety symptoms.

Multivariable linear regression analyses revealed no significant association between sedentary behaviour (daily sitting time) and sleep quality (β = 0.012; 95% CI: −0.313 to 0.337; *p* = 0.941; model R^2^ = 0.301; Figure 6). Similarly, no significant association was observed between sedentary behaviour and anxiety symptoms (β = 0.158; 95% CI: −0.029 to 0.345; *p* = 0.097; model R^2^ = 0.313; Figure 7), suggesting that sedentary time was not associated with either outcome.

### 3.6. Association Between Sleep Quality and Anxiety Symptoms

**Figure 8:**
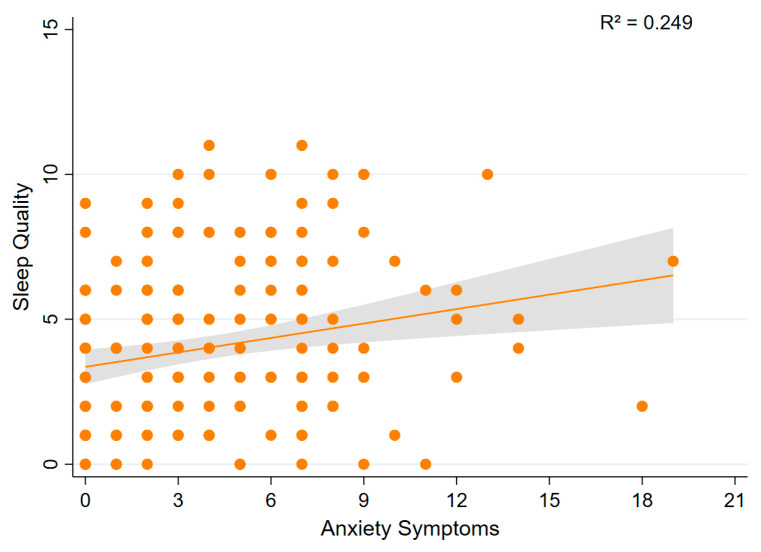
Association between sleep quality and anxiety symptoms. A scatter plot showing the relationship between sleep quality and anxiety symptoms.

Multivariable linear regression analysis showed that anxiety symptoms were significantly associated with poorer sleep quality (β = 0.189; 95% CI: 0.052 to 0.325; *p* = 0.007; model R^2^ = 0.249; Figure 8). This indicates that higher anxiety scores were associated with higher (worse) sleep quality scores.

## 4. Discussion

This study explored the associations between physical activity, sedentary behaviour, sleep quality, and anxiety symptoms among a sample of healthy, pain-free adults in Saudi Arabia. The findings offer multiple important insights. First, although a majority of participants (60%) met the WHO-recommended threshold for physical activity, no statistically significant associations were observed between physical activity levels and either sleep quality or anxiety symptoms. Second, two significant positive associations were identified: one between poor sleep quality and elevated anxiety symptoms and another between increased sedentary behaviour time and higher anxiety symptoms. These findings underscore the potential importance of targeting sleep and sedentary behaviour time in public health efforts to improve mental health outcomes, even in relatively healthy populations.

The absence of statistically significant associations between physical activity and either sleep quality or anxiety symptoms in the present study stands in contrast to a substantial body of international evidence [1,23,25]. Numerous systematic reviews and meta-analyses have consistently demonstrated the beneficial effects of physical activity on both sleep-related parameters, such as sleep efficiency, latency, and duration, as well as on mental health outcomes including anxiety symptoms [23,24]. For instance, Kredlow et al. [23] reported that regular physical activity significantly enhances sleep quality, potentially through mechanisms such as circadian rhythm alignment and hormonal regulation [25,26]. The anxiolytic effects of physical activity have also been extensively documented and are commonly attributed to a combination of physiological and psychological mechanisms [22,23,30,46]. Within the Saudi context, several studies have similarly reported significant positive associations between physical activity and sleep quality [5,11,16,17,31], as well as between physical activity and reduced anxiety symptoms [4,7,16], thereby reinforcing the relevance of these relationships across diverse cultural settings. For example, Mahfouz et al. [5] and Alshammari et al. [16] observed that higher levels of physical activity were associated with better sleep quality among university students. In addition, Al-Raddadi and Al-Dubai [17] and Alkhaldi et al. [31] reported comparable findings among women and the general adult population. Regarding anxiety symptoms, Sayed et al. [7] and Alotaibi and Boukelia [4] found that individuals who engaged in higher levels of physical activity reported significantly lower levels of anxiety during and following the COVID-19 pandemic. These consistent findings across various Saudi subpopulations, including students, women, and the general public, further support the broader international literature affirming the beneficial impact of physical activity on both sleep and anxiety outcomes.

Several plausible factors may account for the divergence between our findings and the broader literature. A primary consideration is the stringent eligibility criteria applied in the present study. Participants were systematically screened to exclude not only individuals with musculoskeletal pain but also those with any self-reported clinically significant health conditions—including serious injuries, major surgeries, or even mild and well-controlled medical conditions such as hypertension, diabetes, or asthma. Consequently, the resulting sample was exceptionally healthy and homogeneous, exhibiting minimal variability in key health indicators. This limited range may have substantially reduced the statistical power to detect associations, particularly when both the predictor (physical activity) and outcome variables (sleep quality and anxiety) demonstrated restricted variance. In addition, the high mean physical activity level observed among participants (1730.02 ± 2109.24 MET min/week) suggests that many individuals were already meeting or exceeding recommended activity levels. This could have introduced a ceiling effect, whereby additional physical activity conferred limited incremental benefits for sleep or mental health—an effect that has been noted in studies examining diminishing returns of exercise among highly active individuals [2,24]. In addition, the high mean physical activity level observed in this sample (1730 ± 2109 MET-min/week), with many individuals exceeding recommended thresholds, may have reduced the variability required to detect stronger associations with sleep quality and anxiety symptoms. Epidemiological evidence suggests that engaging in excessively frequent or intense physical activity (e.g., six or more sessions per week) is associated with greater difficulty initiating sleep, indicating possible diminishing returns or saturation effects at elevated activity levels [47]. Furthermore, findings from experimental and modelling studies indicate that total daily energy expenditure may become attenuated as physical activity increases, due to compensatory physiological adaptations [48,49]. These mechanisms may partially account for the modest associations identified between physical activity and the examined outcomes in the present study. Methodological differences may also contribute to the observed discrepancies, particularly in terms of population characteristics, sample composition, and assessment tools. Another important consideration is the reliance on self-reported data for all primary variables, which introduces the potential for recall and reporting biases. Specifically, participants may have overestimated their physical activity levels, as commonly reported in studies utilising self-administered questionnaires [31,40]. Such misclassification could attenuate true associations. The use of objective measurement tools (e.g., accelerometers for physical activity or actigraphy for sleep) could provide more accurate and sensitive data in future studies. Moreover, including multidimensional assessments of anxiety that capture specific sources of psychological distress, such as perceived stress or situational anxieties, may help elucidate more nuanced relationships between lifestyle behaviours and mental health.

The only statistically significant and robust association identified in this study was between poor sleep quality and anxiety symptoms. This finding is consistent with a substantial body of evidence from both global and Saudi-based research supporting a bidirectional relationship between sleep disturbances and psychological distress. Several physiological mechanisms have been proposed to explain this association, including dysregulation of the hypothalamic–pituitary–adrenal (HPA) axis, increased activity of the sympathetic nervous system, and alterations in REM sleep patterns such as delayed onset or reduced duration [22,24,29]. In the present sample, approximately 30 percent of participants, despite reporting no chronic medical or psychological conditions, exhibited poor sleep quality. This highlights the potential burden of undetected sleep disturbances even among low-risk, apparently healthy individuals. Several studies conducted in Saudi Arabia have similarly demonstrated this association. Al-Khani et al. [14] reported a significant link between poor sleep quality and elevated anxiety symptoms among medical students. Alshammari et al. [16] found that both sleep disturbances and anxiety independently predicted depression among university students. Iqbal et al. [20] observed a strong correlation between anxiety and disrupted sleep patterns during the COVID-19 pandemic, while Alkahtani et al. [19] identified a high co-occurrence of insomnia and anxiety symptoms among the general adult population in Riyadh. These converging findings underscore the importance of routinely assessing sleep quality within mental health evaluations and support the development of integrated intervention strategies targeting both sleep and anxiety symptoms in the Saudi context.

These findings offer several key implications for public health efforts and future research within the Saudi context. First, the significant association between poor sleep quality and elevated anxiety symptoms underscores the importance of routinely assessing sleep during mental health evaluations, even in healthy adult populations. Second, although no significant associations were identified between physical activity or sedentary behaviour and either sleep quality or anxiety symptoms, these behaviours remain important targets for health promotion due to their high prevalence in Saudi Arabia. Culturally relevant strategies, such as improving infrastructure, encouraging active transport, and implementing workplace wellness programmes, may support more active and less sedentary lifestyles. Finally, the results suggest that sleep quality may have a more immediate influence on mental health than physical activity in low-risk populations, highlighting the value of integrated lifestyle interventions that address sleep, anxiety, and activity patterns together.

This study has several limitations. First, the cross-sectional design precludes any inference of causality or temporal ordering among variables. Longitudinal or experimental designs are needed to determine whether improving sleep quality or reducing sedentary behaviour directly leads to reduced anxiety. Second, the reliance on self-reported data, while mitigated by the use of validated instruments, may still introduce recall or reporting bias. In particular, social desirability bias is an inherent consideration in self-administered surveys, whereby respondents may unintentionally overestimate engagement in socially valued behaviours, such as physical activity, or underestimate less favourable behaviours, such as prolonged sedentary time. While the online format facilitated broad and efficient data collection, the absence of direct researcher supervision may contribute to variability in how participants interpret and respond to certain items. Third, the use of convenience sampling through social media platforms facilitated participant recruitment; however, this non-probability approach may introduce selection bias and limit the representativeness of the sample. Furthermore, due to the open nature of social media dissemination, participants could have been indirectly invited by others, making it difficult to assess platform-specific reach, response rates, or drop-off patterns. As a result, accurately evaluating self-selection bias and overall recruitment dynamics was not feasible, which should be considered when interpreting the generalisability of the findings. Fourth, the exclusion of individuals with musculoskeletal pain, any self-reported medical condition (including mild or well-controlled cases), as well as those with serious injuries or major surgeries, enhanced the internal validity of the study by ensuring a homogeneously healthy, pain-free sample. However, this stringent approach also limits the generalisability of the findings to the broader adult population, particularly those with common health conditions or functional limitations. Fifth, the relatively small sample size and overrepresentation of women (nearly 70%) may constrain the applicability of results to men or other demographic subgroups. Future studies should address these issues through larger, more diverse samples, objective measurement tools, and incorporation of additional psychosocial variables.

## 5. Conclusions

This study explored the associations among physical activity, sedentary behaviour, sleep quality, and anxiety symptoms in a specific sample of healthy, pain-free adults residing in Saudi Arabia. The findings revealed that poorer sleep quality was significantly associated with higher levels of anxiety symptoms. No significant associations were found between physical activity or sedentary behaviour and either sleep quality or anxiety. These results underscore the importance of sleep quality as a key behavioural factor associated with anxiety symptoms, even in otherwise healthy adults.

The lack of statistically significant associations between physical activity or sedentary behaviour and sleep quality or anxiety symptoms should be interpreted with caution. Although no significant relationships were observed in this sample, the reliance on self-reported measures may have introduced measurement error or reporting bias, potentially attenuating true associations. Furthermore, the absence of significant findings does not indicate that physical activity and sedentary behaviour are unimportant for sleep or mental health. Rather, the null findings may reflect the complex and multifactorial nature of these relationships, which are influenced by a range of individual and contextual factors that may not be adequately captured within the current cross-sectional study design. In addition, the methodological constraint of restricting the sample to healthy, pain-free adults while excluding individuals with musculoskeletal pain or medical conditions may have reduced variability in key behavioural and psychological measures, potentially influencing the observed associations.

Public health strategies aimed at reducing anxiety in healthy adult populations should prioritise improving sleep quality while continuing to support physical activity and reduced sedentary behaviour as part of a comprehensive approach to well-being. Multicomponent interventions that integrate sleep hygiene, active living, and psychological support may offer greater impact than single-focus strategies. Future research should utilise longitudinal study designs and objective measurement tools, such as accelerometers and actigraphy, to improve the accuracy of behavioural assessments and support stronger causal inferences. Future research should additionally incorporate polysomnography for detailed assessment of sleep architecture and include psychosocial variables such as stress and perceived social support to more fully capture the complex interactions influencing mental health. Additionally, broader and more inclusive sampling strategies are needed to enhance the generalisability of findings and to examine whether these associations differ across populations with varying health conditions, including individuals with musculoskeletal pain or chronic medical conditions.

## Figures and Tables

**Table 1:** Demographic information.

Items		Mean ± SD/Number (%)
Age		33.99 ± 12.57
Gender	Male	56 (30.27%)
	Female	129 (69.73%)
Height (cm)		169.17 ± 9.56
Weight (kg)		75.97 ± 19.94
BMI (kg/m^2^)		26.30 ± 5.53
Nationality	Saudi	169 (91.35%)
	Non-Saudi	16 (8.65%)
Region of Residence	Asir	10 (5.41%)
	Al-Bahah	2 (1.08%)
	Al-Jawf	2 (1.08%)
	Al-Qassim	0 (0%)
	Eastern	17 (9.19%)
	Hail	3 (1.62%)
	Jazan	4 (2.16%)
	Mecca	100 (54.05%)
	Medina	4 (2.16%)
	Najran	2 (1.08%)
	Northern Borders	1 (0.54%)
	Riyadh	37 (20%)
	Tabuk	3 (1.62%)
Marital Status	Single	94 (50.81%)
	Engaged	4 (2.16%)
	Married	82 (44.32%)
	Divorced	4 (2.16%)
	Widowed	1 (0.54%)
Educational Attainment	Less than high school	3 (1.62%)
	High school	49 (26.49%)
	Diploma (student or graduate)	16 (8.65%)
	Bachelor’s degree (student or graduate)	76 (41.08%)
	Master’s degree (student or graduate)	22 (11.89%)
	Doctoral degree (student or graduate)	19 (10.27%)
Current Employment Status	Employed (full-time)	82 (44.32%)
	Employed (part-time)	9 (4.86%)
	Self-employed	5 (2.70%)
	Student only	49 (26.49%)
	Unemployed	25 (13.51%)
	Retired	15 (8.11%)
Primary Work Environment	Office/desk-based work	79 (42.70%)
	Standing/on-foot work	68 (36.76%)
	Physically demanding/manual work	14 (7.57%)
	Mobile/field-based work	12 (6.49%)
Smoking Status	Never smoked	131 (70.81%)
	Current smoker	25 (13.51%)
	Occasional smoker	15 (8.11%)
	Former smoker	14 (7.57%)

**Table 2:** Descriptive statistics of physical activity, sleep quality, and anxiety scores.

Outcome Measure		Mean ± SD
GPAQ	Physical activity (total MET min/week)	1730.02 ± 2109.24
	Sedentary behaviour (total h/day)	6.77 ± 4.69
B-PSQI (total score)		4.05 ± 2.75
GAD-7 (total score)		4.16 ± 3.66

GPAQ: Global Physical Activity Questionnaire; MET: metabolic equivalent task; B-PSQI: Brief Version of the Pittsburgh Sleep Quality Index; GAD-7: Generalised Anxiety Disorder-7 scale.

**Table 3:** Distribution of anxiety severity (GAD-7) across sleep quality (B-PSQI) categories.

Anxiety Severity	Sleep Quality	
	Good Sleep	Poor Sleep
Minimal anxiety	85 (63.91%)	24 (46.15%)
Mild anxiety	41 (30.83%)	23 (44.23%)
Moderate anxiety	6 (4.51%)	4 (7.69%)
Severe anxiety	1 (0.75%)	1 (1.92%)
Total	133 (100%)	4.16 ± 3.66
*p*-value (chi-square test)	0.163	

GAD-7: Generalised Anxiety Disorder-7 scale; B-PSQI: Brief Version of the Pittsburgh Sleep Quality Index.

## Data Availability

The original contributions presented in the study are included in the article; further enquiries can be directed at the corresponding author.

## Supplementary Materials

The following supporting information can be downloaded at https://www.mdpi.com/article/10.3390/sports13070196/s1, Table S1: STROBE Statement—checklist of items that should be included in reports of cross-sectional studies.
